# Consensus statement: Supporting Safer Conception and Pregnancy For Men And Women Living with and Affected by HIV

**DOI:** 10.1007/s10461-017-1777-7

**Published:** 2017-05-13

**Authors:** Lynn T. Matthews, Jolly Beyeza-Kashesya, Ian Cooke, Natasha Davies, Renee Heffron, Angela Kaida, John Kinuthia, Okeoma Mmeje, Augusto E. Semprini, Shannon Weber

**Affiliations:** 1 MGH Global Health and Division of Infectious Diseases, 125 Nashua Street, Suite 722, Boston, MA 02114 USA; 2 Department of Obstetrics and Gynaecology, Mulago National Referral Hospital, Kampala, Uganda; 3 University of Sheffield, Sheffield, UK; 4 University of the Witwatersrand, WITS RHI, Johannesburg, South Africa; 5 University of Washington, Seattle, USA; 6 Faculty of Health Sciences, Simon Fraser University, Burnaby, British Columbia Canada; 7 University of Washington, Kenyatta National Hospital, University of Nairobi, Nairobi, Kenya; 8 University of Michigan, Ann Arbor, USA; 9 University of Milan, Milan, Italy; 10 University of California at San Francisco, Zukerberg San Francisco General Hospital, San Francisco, USA

## Abstract

Safer conception interventions reduce HIV incidence while supporting the reproductive goals of people living with or affected by HIV. We developed a consensus statement to address demand, summarize science, identify information gaps, outline research and policy priorities, and advocate for safer conception services. This statement emerged from a process incorporating consultation from meetings, literature, and key stakeholders. Three co-authors developed an outline which was discussed and modified with co-authors, working group members, and additional clinical, policy, and community experts in safer conception, HIV, and fertility. Co-authors and working group members developed and approved the final manuscript. Consensus across themes of demand, safer conception strategies, and implementation were identified. There is demand for safer conception services. Access is limited by stigma towards PLWH having children and limits to provider knowledge. Efficacy, effectiveness, safety, and acceptability data support a range of safer conception strategies including ART, PrEP, limiting condomless sex to peak fertility, home insemination, male circumcision, STI treatment, couples-based HIV testing, semen processing, and fertility care. Lack of guidelines and training limit implementation. Key outstanding questions within each theme are identified. Consumer demand, scientific data, and global goals to reduce HIV incidence support safer conception service implementation. We recommend that providers offer services to HIV-affected men and women, and program administrators integrate safer conception care into HIV and reproductive health programs. Answers to outstanding questions will refine services but should not hinder steps to empower people to adopt safer conception strategies to meet reproductive goals.

## Context and Purpose

Globally, at least 20–50% of men and women living with HIV want to have children [1–4]. For many HIV-affected individuals and couples, attempting pregnancy introduces risks of HIV transmission to infants and HIV-uninfected partners. Antiretroviral-driven HIV-prevention strategies reduce risks of HIV transmission through pregnancy attempts and pregnancy. Programs to prevent or eliminate maternal-to-child-transmission (PMTCT/EMTCT) reduce perinatal transmission to less than 2% for women living with HIV [5]. Antiretroviral therapy (ART) extends the health and survival of people living with HIV (PLWH) to a life expectancy equivalent to that of HIV-negative persons [6, 7]. Furthermore, HIV prevention programs offer multiple approaches to reduce sexual HIV transmission risk during condomless sex [8, 9]: these can be promoted when people affected by HIV desire and attempt pregnancy.

A growing body of evidence suggests opportunities for widespread promotion of safer conception counseling and service delivery. Safer conception interventions reduce HIV incidence while supporting the pregnancy goals of men and women living with or affected by HIV. Safer conception program outputs dovetail with global goals to enhance HIV testing, ART uptake, and HIV-RNA suppression, and thus reduce HIV incidence and eliminate perinatal transmission. However, a limited number of country-level policies and a lack of global guidelines leave providers unprepared to counsel clients. Therefore, grounded in a reproductive rights-based framework, with foundations in scientific data and community values, we developed this consensus statement to: articulate experiences with and address growing demand for safer conception services; summarize the science; identify information gaps; outline research and policy priorities; and advocate for action.

### History of Growing Consensus on Safer Conception

This consensus statement was motivated by discussions over the last decade. Safer conception research, policy, and implementation has been highlighted at international meetings [10–15] [16–18], leading journals have highlighted research in this field through supplements [19] [20, 21] [22], and several organizations advocate for and provide safer conception care for persons-affected by HIV [23] [24] [25]. The Global Network of People Living with HIV [15] and the International Community of Women Living with HIV [26] endorse safer conception programming and sexual and reproductive health and rights for people living with or affected by HIV. Canada [27, 28], South Africa [29], and the UK [30]) have national guidelines offering comprehensive safer conception guidelines.

### Call for Consensus Document

The call for this global consensus statement to advocate for safer conception implementation emerged from a satellite session at the International AIDS Society 2015 Conference titled “Achieving pregnancy while minimizing HIV transmission risks: Safer conception research, policy, and programming priorities for HIV-affected individuals and couples” [31]. This session convened approximately 180 stakeholders from 25 countries including PLWH, clinicians, researchers, policy makers, community advocates, and donor agencies.

### Our process

This document emerged from a consultative process incorporating input from the above meetings, literature, and discussions with key stakeholders. Three co-authors (LTM, RH, AK) developed a draft outline of the manuscript which was discussed and modified with co-authors and other experts in safer conception, HIV treatment and prevention, fertility, and perinatal transmission.

The updated outline informed a draft of the statement and feedback was sought from co-authors and key stakeholders (consumers, persons living with HIV, research and clinical leaders in safer conception). Feedback was integrated to develop a penultimate draft of the manuscript. All co-authors approved the final draft of the consensus guidelines. Subsequently, the statement was posted online and circulated to key stakeholders including organizations, clinicians, community members, researchers, and advocates in sexual and reproductive health, fertility, and HIV for endorsement (https://www.hiveonline.org/saferconceptionendorse/).

## Consensus

We summarize the evidence regarding demand for, strategies for, and implementation of safer conception services. Several related topics such as contraception, antiretroviral treatment choices during pregnancy, perinatal transmission of HIV, and breastfeeding practices are not explicitly covered in this document. We outline key questions that remain unanswered and suggest steps for the way forward. We articulate the need for action to provide services to minimize pregnancy-related HIV transmission risks while supporting the sexual and reproductive goals of men and women affected by HIV. We urge providers and policy-makers to support safer conception service implementation even as research and advocacy continue.

### Demand for Safer Conception Services: Evidence

For most men and women, an HIV diagnosis does not eliminate hopes or plans to have children, yet few access safer conception care [32–35]. Individual stories articulated by people living with HIV highlight the demand and opportunities for service delivery (See Box 1).

**Box 1:** Pregnancy experiences of HIV-affected men and women

*Jacque* is a Kenyan woman in her early 40 s. She was diagnosed with HIV in 2004. 5 years after learning her positive HIV status, she discovered she was pregnant again. Although this was an unplanned pregnancy, it was not unwanted. Her partner at the time was also living with HIV. The reaction of her community as they learned that she was a pregnant woman living with HIV surprised her. They questioned why a woman living with HIV would become pregnant and accused her of wanting to give her unborn child AIDS. Nonetheless, she carried on with the pregnancy, received PMTCT care, and was blessed with a son who is now 7 years old and HIV-negative. However, Jacque’s experiences of stigma and discrimination throughout her pregnancy caused her to limit her desire to expand her family further, until recently She now has a partner who is HIV-negative and he would like to have a child with her. He is concerned about acquiring HIV and the risks to a future baby. He does not know if it is possible to conceive with an HIV-positive partner and anticipates facing judgment from his family, community, and healthcare providers. As an activist and well-informed woman, Jacque knows that safer conception is possible but feels the burden of responsibility to find a willing provider or take it upon herself to educate and inform her HIV care providers to support the fertility desires she and her partner now have
*Juan* ^a^ is a North American man living with HIV. When he was diagnosed with HIV in 2008, he worried about whether he would be able to have a family. Juan’s provider asked him about whether or not he wanted a child in the future, discussing the options together. When Juan began dating Monica, she came to clinic with him and learned about reducing HIV transmission, including pre-exposure prophylaxis/PrEP. Monica chose to take PrEP and stay on contraception until she finished school. After graduating, she continued PrEP and became pregnant. Monica remains HIV-negative and delivered an HIV-negative baby. Juan remains engaged in care and maintains an undetectable HIV-RNA viral load
*Lisa* is a young Canadian woman who acquired HIV infection via perinatal transmission. As a young child, she received HIV care including antiretroviral therapy through a local clinic for HIV-infected children, women, and their families. In her 20 s, Lisa fell in love with and married an HIV-negative man. When the couple decided to expand their family, they sought guidance from Lisa’s HIV providers, with whom she had developed a trusting relationship. Lisa’s care providers supported her pregnancy plans and offered the couple safer conception counseling. Lisa successfully used ART and had an undetectable viral load for at least six months before the couple started having sex without condoms to try to get pregnant. They paid attention to time their condomless sex to days with high fertility. Within a year, Lisa and her husband welcomed a baby girl. Both her husband and baby remain HIV-uninfected. Lisa credits her supported journey to motherhood to her HIV care providers’ commitment to integrated, women-centered, and holistic management of HIV, from diagnosis, treatment initiation, and conception, to pregnancy, postpartum, and beyond

Stories articulated by the men and women represented above. Edited by the authors

^a^ Pseudonym

The demand, opportunities, and challenges expressed in these stories echo throughout the literature. Most men and women living with or affected by HIV have limited awareness about HIV prevention options that reduce transmission during condomless sex [35, 36]. Providers do not initiate conversations about reproductive goals with HIV-affected clients [33, 35, 37–39] and anticipated stigma from providers is a barrier to seeking care [34, 40, 41]. In addition, individuals and couples who want to have children may not explicitly plan for pregnancy, limiting opportunities for pre-conception counseling [35, 42, 43]. HIV counseling and testing can be an entry point to discussions about pregnancy plans and safer conception care but uptake of pre-conception HIV testing is low [44]. Furthermore, this is not a one-time conversation, as reproductive goals evolve over time. For many seeking pregnancy, reproductive services and/or fertility evaluation may be necessary, but ongoing stigma and limited resources constrain access to fertility services for PLWH. Among those who know their serostatus, barriers to disclosure to sexual partners and confusion about serodiscordance limit recognition that conception attempts may be accompanied by HIV exposure [40, 45, 46]. When discussion about safer conception is started, provider guidance about the various methods is desired [47, 48]. Men’s voices are nearly absent in conversations about sexual and reproductive health for persons living with or affected by HIV [49]. Furthermore, many men and women make decisions and plans regarding reproductive goals together with families and communities where knowledge of safer conception opportunities is low. See Table 1 for summary consensus points in the area of Demand for Safer Conception Services.

**Table 1:** Summary group consensus points based on the available evidence

Demand for safer conception services	∙ There is widespread demand for safer conception services. In the absence of support for informed decisions about safer conception practices, HIV-affected individuals and couples navigate pregnancy decisions without information to support HIV risk reduction opportunities ∙ Stigma towards PLWH having children limits access to comprehensive reproductive care ∙ Insufficient knowledge, a lack of normative safer conception guidance, and limited skills regarding safer conception options prevent healthcare providers from initiating conversations about reproductive goals with PLWH and their partners ∙ Attrition across the HIV care cascade limits the reach and potential of safer conception care. Efforts are required to ensure that HIV prevention services are available to all who require them ∙ Reproductive goals, risk perceptions, and acceptance of both risks and risk reduction strategies are fluid and thus not readily addressed by one-time counseling ∙ Safer conception information needs to be shared beyond individual-level clinic counseling to include partners, family members, and communities.
Opportunities for HIV prevention in the context of desired pregnancy	∙ There is clear scientific evidence regarding efficacy, effectiveness, safety, and client acceptability supporting a range of biomedical and behavioral safer conception strategies ∙ Antiretroviral treatment (ART) for people living with HIV is indicated for their own health, and also to reduce transmission to partners and (for women) infants at all times, including when pregnancy is intended ∙ Choices provide options and engage people living with HIV in care. PrEP for HIV-negative partners, limiting condomless sex to peak fertility, home insemination, male circumcision, treatment of sexually transmitted infections, couples-based HIV testing, semen processing and assisted reproductive technologies, and fertility care provide additional HIV prevention options to support individual preferences and the realities of accessible services
Implementation of comprehensive sexual reproductive health services	∙ Safer conception service implementation is limited by a lack of guidelines, a lack of service, delivery and population targets, and a lack of training and support for providers to offer these services ∙ Many providers have ongoing reservations about supporting condomless sex and/or childbearing among PLWH ∙When services are made available, demand is high, delivery is feasible, and outcomes are encouraging ∙ Supporting mutual HIV-serostatus disclosure within the partnership is part of safer conception counseling, but disclosure to a potential pregnancy partner is not a prerequisite for participation ∙ Legal and ethical issues present challenges to the delivery of safer conception care. Potential clients should be counseled about relevant legal considerations. However, refusal to provide safer conception services violates the reproductive rights of PLWH

### Opportunities for HIV Prevention in the Context of Desired Pregnancy: Evidence

Several strategies allow HIV-affected couples to conceive with minimal transmission risk to the uninfected partner (Table 2). All PLWH, regardless of reproductive plans, are encouraged to initiate ART upon HIV diagnosis to improve their own health, reduce morbidity and mortality, and reduce HIV transmission [50]. When pregnancy is desired, the benefits of ART extend to safer conception. It is ideal for individuals and couples to delay pregnancy attempts until HIV-RNA is suppressed or after at least six months of ART [51, 52].

**Table 2:** Strategies to reduce periconception risk of HIV transmission for serodiscordant couples

Couple	Method	Estimated risk reduction	Level of evidence^a^ [source]
Either partner infected, pursuing sex without condoms for pregnancy + adjunct risk reduction strategies (goal: $\downarrow$ sexual transmission)	Sex without condoms limited to peak fertility	Unknown	1A [96]
	ART for the infected partner	96%	1B [52, 97]
	PrEP (oral, daily FTC/TDF or TDF) for the uninfected partner	63–75%	1A^b^ [98, 99]
	Post-exposure prophylaxis (PEP) for the uninfected partner	Unknown	2A
	Treatment of STI’s	≤40%	1B^c^ [60, 61]
F + M- (goal: $\downarrow$ female to male transmission)	Manual self insemination^e^	100%	5 [58]
	Medical male circumcision	66%	1A [59]
M + F- (goal: $\downarrow$ male to female transmission)	Sperm washing^d^	~100%	2A [64]

*IUI* intrauterine insemination,* IVF* in vitro fertilization,* ICSI* intracytoplasmic sperm injection,* ART* antiretroviral treatment,* PrEP* pre-exposure prophylaxis,* FTC/TDF* emtricitabine/tenofovir disoproxil fumarate

^a^ Oxford Centre for Evidence-based Medicine, Levels of Evidence (1A: RCT’s with homogeneous support; 1B: individual RCT; 2A: cohort studies with homogeneity; 2C: ecological studies; 5: expert opinion without explicit supporting research) [100]

* Limiting sex without condoms to times of peak fertility reduces exposure, but does not affect HIV-1 transmission risk per coital act

^b^ Effective for heterosexual men in two of two RCTs and for women in two of four RCTs

^c^ Effective in one of six RCTs

^d^ Sperm washing can be followed by introduction to the female partner via cervical cap, IUI, IVF ± ICSI, depending on available services, client preference, and concerns re. fertility. Additional strategies that support building of healthy families for HIV-affected couples include donor sperm and adoption

^e^ Man ejaculates into a condom or cup and the contents are introduced via condom reversal or needleless syringe at home, or through IUI with a healthcare professional - timed to the woman’s peak fertility

Because uptake of and adherence to ART is often imperfect or unknown to the partner, individuals may choose additional methods to reduce transmission risk. Pre-exposure prophylaxis (PrEP) is recommended for those at substantial risk of HIV and is an important option for an uninfected partner wishing to conceive with an infected partner whose ART use is suboptimal or unknown [50]. For men or women exposed to HIV and not yet on PrEP, antiretroviral post-exposure prophylaxis (PEP) may reduce HIV acquisition risks and can be offered within 72 h of exposure and continued for 28 days after exposure [53] [50]. For individuals or couples exposed to HIV as part of conception attempts, PEP may be a bridge to PrEP [42]. When used prior to pregnancy, there are minimal safety or fertility concerns about tenofovir-based PrEP [54, 55]. For uninfected women, safety data about TDF/FTC-based PrEP during pregnancy are based on studies of women living with HIV or hepatitis B exposed to combination ART throughout pregnancy. The data are largely reassuring but inconsistent results suggest possible decreased bone mineral density in newborns, with unknown clinical significance, and one study showed risk of adverse infant outcomes (low birth weight or even stillbirth) among infants exposed to TDF/FTC as part of ART, a finding not replicated in other studies [55, 56]. This warrants additional data collection among women using PrEP throughout pregnancy. Current guidelines recommend PrEP as a safer conception strategy with discussion of the risks and benefits of continuing PrEP use during pregnancy and breastfeeding [50, 57].

Additional strategies to decrease HIV acquisition by men during pregnancy attempts with a woman living with HIV include vaginal self-insemination, which eliminates the need for condomless sex [58]; and medical male circumcision [59]. Treatment for sexually transmitted infections (STIs) has been shown to further reduce HIV transmission risk [60, 61]. Limiting condomless sex to days with peak fertility [62] maximizes likelihood of achieving pregnancy while limiting HIV exposure.

For HIV-serodiscordant couples where the man is living with HIV, sperm processing or “washing” can isolate sperm (which do not harbor HIV) from seminal plasma and leukocytes [63]. Processed semen can be introduced via intrauterine insemination, in vitro fertilization with or without intracytoplasmic sperm injection, or via a cervical cap. Robust data suggest that this process is safe and effective, with no recorded HIV transmissions to pregnancy partners [64]. This is an important option but should by no means be mandated [65].

Compromised fertility among HIV-affected couples may result in HIV-exposure with minimal chances of pregnancy [66]. Infertility screening may identify couples who would benefit from services to optimize fertility prospects prior to pregnancy attempts [67]. Comprehensive safer conception care also includes offering contraception until couples are ready to attempt pregnancy and/or once they have met their reproductive goals. See Table 1 for summary consensus points in the area of “Opportunities for HIV prevention in the context of desired pregnancy.”

### Implementation of Comprehensive Sexual Reproductive Health Services: Evidence

#### Normative Guidance

Few countries have safer conception clinical guidelines. Canada, South Africa, and the UK have guidelines; however, many providers in these locations remain unprepared to counsel clients [35–37, 39, 41, 68, 69]. The WHO recommends ART for all PLWH and PrEP for HIV-exposed persons. Current global EMTCT guidance does not explicitly address safer conception programming.

#### Systems

Most safer conception services exist within specialized academic medical centers, private clinics, research projects, or privately funded implementation programs [42, 70–73]. Data show that where services are offered, uptake is high and outcomes are encouraging. These programs provide opportunity to inform future implementation efforts.

#### Reproductive Rights of People Living with HIV

A reproductive rights framework recognizes that all couples and individuals, including those living with or affected by HIV, have the basic right to “decide freely and responsibly the number, spacing, and timing of their children and to have access to the information, education, and means to do so” [74]. These rights also include the right to make reproductive decisions “free of discrimination, coercion, and violence”. The endorsement of the sexual and reproductive rights of PLWH is essential within safer conception guidelines, policies, and programs as a step towards building trust between providers and HIV-affected clients, given a history of sexual and reproductive rights violations of PLWH [75].

Legal and ethical issues present challenges to safer conception care delivery. Expectations that clients seeking pregnancy disclose HIV status to pregnancy partners must be considered in the context of clients’ lives and safety. Supporting safe disclosure is a component of safer conception programming, but not a prerequisite for participation. For many PLWH, particularly women, disclosure is highly challenging and in some cases dangerous—with risks of violence, relationship dissolution, and abandonment [76]. Disclosure challenges are exacerbated in settings where laws criminalize HIV non-disclosure, exposure, and/or transmission to sexual partners [77]. Institutionalized stigma and HIV criminalization are likely to influence willingness of HIV-affected individuals to engage and remain in HIV care [78] and safer conception care.

#### Providers

Providers rarely counsel clients about safer conception options [33, 35–39, 41, 68, 79–81]. In addition, many providers retain negative attitudes towards PLWH having children, in part due to experiences caring for children living with HIV, historical recommendations against pregnancy for PLWH, and lingering stigma and discrimination [36, 37, 39, 41, 81, 82]. After decades of focusing on condoms for HIV prevention, providers hesitate to endorse condomless sex [41, 81]. Provider time is limited and in demand. In addition, clients who want safer conception advice often hesitate to ask for it, in part due to the emphasis on condom use and perceptions of provider stigma towards pregnancy among PLWH [34, 40, 41, 82]. HIV-affected men and women may also seek care from traditional healers and other providers who may not have safer conception information [83, 84]. See Table 1 for summary points regarding Implementation of Comprehensive Safer Conception Services.

### Key Questions Within the Areas of Demand, Opportunities for Prevention, and Implementation

We identified key questions within the areas of demand, opportunities for prevention, and implementation. Answers to these questions will improve care and implementation and inform policy, but need not delay service implementation.

#### Demand

How to reach at-risk, uninfected men and women who may benefit from safer conception services as well as PLWH not engaged in care or aware of their own or their partner’s serostatus is a gap. Normalizing pregnancy desires and pregnancy among people living with and affected by HIV is an outstanding goal to decrease stigma.

#### Prevention

The science of HIV prevention offers clear data regarding reduced risk of sexual transmission of HIV, but few methods have been studied in the context of couples or individuals seeking personal or partner pregnancy. Research to understand method use in the context of desired pregnancy is needed. While HIV-RNA suppression would ideally be confirmed prior to conception attempts, this is less feasible in resource-limited settings. 6 months is sufficient for the majority of clients to suppress HIV-RNA while taking effective ART. Strategies to test for suppression sooner for those eager to move forward with pregnancy attempts, or for those who feel more comfortable proceeding with proof of HIV-RNA suppression, are needed.

Tenofovir-based PrEP is a relatively new prevention technology and, accordingly, there are ongoing questions. A major challenge is how to best support adherence to PrEP [85]. In addition, there remain outstanding questions regarding the safety of TDF/FTC as prophylactic medication during pregnancy and breastfeeding [86]. Current WHO guidelines recommend PrEP as a safer conception strategy with discussion of the risks and benefits of use during pregnancy [50]—but some regional guidelines caution against use in pregnancy [87, 88]. Data to answer these questions are being collected [42, 72, 89]: how to counsel clients and providers to make informed decisions based on available and evolving data is needed [90].

#### Implementation

In early implementation studies, many clients seeking safer conception services have a medical history suggestive of infertility [91, 92]. How to support HIV-affected couples who have compromised fertility is a significant gap. Fertility evaluation after 6 months of timed unprotected sex has been recommended for HIV-negative women older than 35 years [93]. A similar approach has been advocated to minimize the duration of HIV exposure for HIV-affected couples, but there is not consensus about whether a fertility evaluation should be conducted prior to the couple’s attempts at achieving pregnancy, or at six or 12 months after attempted conception. A lack of available resources for fertility assessment and management should not preclude safer conception care [94] (Table 3).

**Table 3:** Resources

Source	Link	Description
Canadian HIV pregnancy planning guidelines (2012)	http://www.jogc.com/article/S1701-2163(16)35274-4/pdf http://www.ncbi.nlm.nih.gov/pubmed/23125998	Comprehensive safer conception guidelines
South African National contraception and fertility planning policy and service delivery guidelines (2012)	http://www.doh.gov.za/docs/policy/2013/contraception_fertility_planning.pdf https://www.health-e.org.za/wp-content/uploads/2014/05/ContraceptionPolicyServiceDelGuidelines2013.pdf	Safer conception guidelines for South Africa (preceding approval of PrEP in SA)
U.S. DHHS Guidelines, Panel on Treatment of HIV-Infected Pregnant Women and Prevention of Perinatal Transmission,	http://aidsinfo.nih.gov/contentfiles/lvguidelines/perinatalgl.pdf	Safer conception methods for HIV-serodiscordant couples are mentioned
ASRM guidelines on offering assisted reproductive technologies to HIV-affected individuals and couples	https://www.asrm.org/uploadedFiles/ASRM_Content/News_and_Publications/Ethics_Committee_Reports_and_Statements/hivethics.pdf http://www.reproductivefacts.org/globalassets/asrm/asrm-content/newsand-publications/ethics-committee-opinions/human_immunodeficiency_virus_and_infertility_treatment-pdfmembers.pdf	Ethics Committee of the American Society for Reproductive Medicine, statement on the safety and ethics of offering assisted reproductive technologies to men and women living with and/or affected by HIV
WHO, Guidelines for use of antiretrovirals (2015)	http://www.who.int/hiv/pub/guidelines/earlyrelease-arv/en/	Guidelines re. the use of antiretrovirals as treatment and prevention
HIVE	https://www.hiveonline.org/	Client-centered website committed to advancing reproductive and sexual wellness for individuals, families and communities affected by HIV in San Francisco and beyond
GlobalShare Listserv	https://www.hiveonline.org/resources/global-share/	GlobalSHARE Google Group is an easily accessible venue to ask and answer important clinical questions, connect individuals to care, share protocols and tools, circulate important papers and presentations, advertise educational and funding opportunities, and create a space to recognize important work being done in the area of safer conception in the context of HIV

Safer conception options are required for those who are not ready for ART, who do not know partner HIV status, or whose partner is not engaged in care, on ART, or adherent. The consensus group agrees that combinations of methods are likely best, but which combinations and how many methods depends on the level of HIV risk, preferences of the individuals, and available services. The importance of balancing a public health approach (which may find PrEP cost-ineffective on top of ART for an infected partner) with personal risk assessment (for example, an individual may not feel confident that his or her partner is adherent to ART) is important to supporting clients while also attending to cost-effectiveness.

Questions regarding implementation are abundant. While ideally a diverse array of providers in HIV-endemic settings should provide these services, the reality is that services will first be offered by a smaller cadre of interested, motivated, or externally-funded providers. How to expand beyond providers with a particular interest in these services remains unclear. Community health workers may play an important role. How to integrate safer conception methods into existing paradigms (e.g. HIV care, sexual and reproductive health care) to leverage resources and maximize efficiencies is unknown. Provider tools that support provision of safer conception counseling and address stigma towards PLWH having children are also needed [82]. We also identified a need for systems to support providers offering care in settings where condomless sex without HIV disclosure among PLWH is criminalized [77, 78].

On the client level of implementation, how best to support safer conception programming for HIV-concordant-positive couples, men, same-sex couples, single parents by choice, extended and co-parenting families are outstanding questions. Ensuring that pregnancy planning and reproductive agency and autonomy are promoted within safer conception services and that providers support clients who want to have children but are not yet able to safely disclose to their partner, are outstanding gaps. How to create consumer-friendly services and tools is an area for future research.

## Conclusions/Recommendations

It is time to implement safer conception services. This consensus is supported by science, consumer demand, and global goals to eliminate perinatal HIV transmission. We recommend that providers offer available safer conception services to HIV-affected men and women, and health program administrators integrate safer conception services into existing HIV and reproductive health programs. While the range of service choices may vary across settings, in all settings a minimal set of safer conception services is available. The most fundamental tenets of safer conception counseling are know your HIV-serostatus, know your partner’s HIV-serostatus, and wait until the partner living with HIV is on effective ART before attempting pregnancy. These basic tenets should be widely shared with HIV-affected persons, families, communities, and providers. We maintain that this work no longer needs to be the purview of specialists. Answers to outstanding questions will refine care, implementation, and policy but do not need to be resolved prior to offering services.

HIV-related stigma is a key barrier to uptake and delivery of safer conception care. People living with and affected by HIV who consider pregnancy or become pregnant and have children express internalized stigma and describe experiences of stigma from providers and community. Stigma compromises the willingness of clients to seek care and provider willingness to address reproductive goals with clients. It is imperative to acknowledge and support the sexual and reproductive rights of persons living with and affected by HIV and focus efforts on reducing stigma at individual-, provider-, and community-levels, such that HIV-affected men and women can make informed decisions and build healthy families.

We must develop tools that support HIV-affected persons to identify potential risk, engage with safer conception services, and use methods that align with preferences and level of risk. The goal is to engage all populations who could benefit from services including mutually-disclosed HIV-serodiscordant heterosexual couples, men and women living with or exposed to HIV, same sex couples, and seroconcordant-positive couples. Safer conception care must be flexible and address a diversity of clients.

We support development and implementation of strategies that can be adopted in diverse settings to support HIV-affected persons to achieve reproductive goals including increased access to ART and PrEP, HIV-RNA testing, STI testing, sperm washing and assisted reproductive technologies, and fertility care. Programs must be nimble in incorporating discussions about reproductive goals at many points in a client’s care without creating undue burden on providers or systems. Integrating safer conception messaging into public health targets for HIV prevention and treatment can maximize synergies.

Provider-initiated conversation is an important gateway to practicing safer conception. Providers across diverse disciplines and training-levels should be able to discuss reproductive options with HIV-affected couples and individuals. Tools to support providers to offer services are required, as are strategies and financing to support service integration [95].

Documenting the effect of safer conception is paramount for guidelines and for the confidence of providers and consumers. EMTCT and treatment as prevention initiatives dovetail with safer conception efforts. Data highlighting the advantages of these synergies may catalyze changes in policy and programs. Ensuring that the voices, values, preferences, and experiences of PLWH are at the forefront of safer conception messaging can normalize care-seeking and pregnancy in the context of HIV.

It is time to seize the opportunity to empower people affected by HIV to embrace their fertility goals and utilize safer conception strategies to satisfy goals for pregnancy with minimal HIV transmission risks. This group consensus is strongly supported by science, consumer demand, and global goals to reduce HIV incidence and eliminate perinatal transmission. We invite you to endorse this statement at https://www.hiveonline.org/saferconceptionendorse/.

## Key Terminology

Safer conception strategies or programs: Strategies or programs that support people living with HIV and/or their partners to achieve pregnancy with minimal risks of sexual transmission of HIV
PLWH, WLWH, MLWH: Persons or person living with HIV, women or woman living with HIV, men or man living with HIV
HIV-affected couple: A couple or partnership in which at least one member of the partnership is living with HIV
HIV-serodiscordant couple or partnership: A couple or partnership in which one member of the partnership is living with HIV and the other is HIV-negative
